# A Novel Role of Globular Adiponectin in Treatment with HFD/STZ Induced T2DM Combined with NAFLD Rats

**DOI:** 10.1155/2014/230835

**Published:** 2014-02-06

**Authors:** Hong Ma, Guo-Ping You, Xuan-Pu Zhang, Xiang-Jiu Yang, Hua-Dong Lu, Yan-Ling Huang, Wen-Qiang Zhang

**Affiliations:** ^1^Department of Endocrinology, Zhongshan Hospital Xiamen University, 209 Hubin South Road, Xiamen, Fujian 361004, China; ^2^Department of Endocrinology, Xiamen Zhongshan Teaching Hospital of Fujian Medical University, Xiamen, Fujian 361004, China; ^3^Department of Pathology, Zhongshan Hospital Xiamen University, Xiamen, Fujian 361004, China

## Abstract

*Aims*. To evaluate the effects of globular adiponectin (gAd) on treatment of type 2 diabetic rats combined with NAFLD. *Materials and Methods*. Twenty-one male wistar rats were fed with normal diet (7 rats) or high fat diet (HFD) (14 rats) for 4 weeks, and then HFD-fed rats were injected with streptozotocin (STZ) to induce type 2 diabetes mellitus (T2DM). Half of T2DM rats were randomly injected with gAd intraperitoneally for 7 days. The expressions of adiponectin receptors (adipoR1/R2) in liver and skeletal muscle tissues were detected through western blotting or RT-qPCR, respectively. *Results*. Globular adiponectin alleviated the hepatic steatosis and increased insulin secretion. In liver, both the protein and mRNA expressions of adipoR2 in T2DM group decreased (*P* < 0.05, resp.) in contrast to NC group and increased (*P* < 0.05 and *P* < 0.001, resp.) after gAd treatment. But the protein and mRNA expressions of adipoR1 increased (*P* < 0.05, resp.) in T2DM group and no change was found in the gAd-treated group. In skeletal muscle, the protein and mRNA expressions of adipoR1 and adipoR2 were upregulated in T2DM group and were downregulated after gAd treatment. *Conclusions*. Globular adiponectin could ameliorate the hepatic steatosis and vary the expressions of adiponectin receptors in liver and skeletal muscle by stimulating insulin secretion.

## 1. Introduction

The occurrence of NAFLD in type 2 diabetes mellitus (T2DM) patients is reported to range from 50% to 75% [1, 2]. NAFLD not only increases the risk of T2DM, but also worsens glycemic control and contributes to the pathogenesis of major chronic complications of diabetes [3]. Presently, there is no consensus on treatment of NAFLD. Most interventions evaluated for the treatment of NAFLD are those commonly used for the treatment of T2DM and they exerted an indirect effect through improvement in insulin resistance (IR).

Adiponectin is a 30 KDa protein, secreted specifically and abundantly in adipose tissue, which has anti-inflammatory, antidiabetic, and antiatherogenic properties [4]. It was confirmed that the level of adiponectin in serum was decreased under conditions of obesity, insulin resistance, NAFLD, and T2DM [5–7]. Accordingly, adiponectin-deficient mice exhibited insulin resistance and diabetes [8]. Conversely, administration of adiponectin included transgenic overexpression of adiponectin or injected recombinant adiponectin prevented development of diabetes and hyperlipidemia [9–11]. Hence, it had been revealed that adiponectin played an important role in the progression of diabetes.

Biological functions of adiponectin depend on not only the serum circulating concentration of hormone but also the expression level and function of its specific receptors (including adipoR1 and adipoR2). AdipoR1 and adipoR2 are ubiquitous; adipoR1 is most preferentially expressed in skeletal muscle and adipoR2 is most preferentially expressed in liver [12]. Some evidences supported that deficiency of adiponectin receptors caused hyperglycemia and hyperinsulinemia [13, 14]. Knockout mice lacking adipoR1 and adipoR2 [15] exhibited loss of the metabolic actions and effects of adiponectin and showed an increased tendency of tissue triglyceride content, inflammation, oxidative stress, insulin resistance, and glucose intolerance. The activation of adipoR1 and adipoR2 resulted in increased hepatic and skeletal muscle fatty acid oxidation, increased skeletal muscle lactate production, reduced hepatic gluconeogenesis, increased cellular glucose uptake, and inhibition of inflammation and oxidative stress [12]. It indicated that adipoR1 and adipoR2 were the predominant mediators of the metabolic effects of adiponectin and they played an important role in transmitting the signal of adiponectin. Nevertheless, the changes of adiponectin receptors stimulated by various factors in adiponectin signal path were controversial. It seemed that both adpoR1 and adipoR2 were with similar patterns to transmit the signal of adiponectin. But Bjursell et al. [16] showed that adipoR1 and adipoR2 were clearly involved in energy metabolism but had opposing effects. The expressions of adipoR1 and adipoR2 in liver, skeletal muscle, and fat were differentially displayed in genetically modified animal models of obesity and diabetes, such as ob/ob and db/db mice [13, 17].

The globular and full length forms of adiponectin exhibit different affinities for two adiponectin receptor (AdipoR1/R2) isoforms [18] and have been shown to mediate distinct effects [9, 19]. The globular adiponectin had been reported to exert more potent effects [20]. Hence, it is suggesting that the recombination globular adiponectin may have a potential effect in the treatment of T2DM, especially combined with NAFLD.

Therefore, we aim to evaluate the effects of globular adiponectin in treatment of type 2 diabetic combined with NAFLD rats induced by high-fat/STZ and further explore the interaction between gAd and adipoR1/R2 in liver and skeletal muscle.

## 2. Materials and Methods

### 2.1. Experimental Model

Twenty-one male adult wistar rats (190–210 grams) supplied by Hayes Lake experimental animals company (Shanghai, China) were acclimatized in communal cages at 25 ± 2°C with a 12 h light and 12 h dark cycle for 1 week with normal diet. Then the rats were randomly divided into three groups, NC group: normal control rats (*n* = 7), T2DM group: type 2 diabetic control rats (*n* = 7), and gAd-treatment group: type 2 diabetes rats treated with gAd (*n* = 7). At the beginning, NC group rats were fed with normal diet, while those of T2DM and gAd-treated group were fed with a high-fat diet (10% fat, 10% carbohydrate, 5% cholesterol, and 75% basis diet) provided by the Animal Experimental Center of Xiamen University.

After 4 weeks, rats of T2DM group and gAd-treated group were intraperitoneally injected with a freshly prepared solution of streptozotocin (STZ; 28 mg/kg, Sigma, St. Louis, MO, USA) in 0.1 M citrate buffer (PH 4.21) to induce type 2 diabetic model compared to those of NC group which were administered with an equal volume of 0.1 M citrate buffer. After STZ injection for 72 h, fourteen rats with random blood glucose level above 16.7 mmol/L were considered as diabetic. Then, seven type 2 diabetic rats were selected randomly into gAd-treated group and were injected intraperitoneally with gAd (BioVision, CA, USA) at a dose of 3.5 ug daily at 9 a.m. for one week, while NC and T2DM group rats received an equal volume of 0.9% saline, respectively. All rats were euthanized at the end of globular adiponectin or 0.9% saline intervention.

All experiments were approved by the Laboratory Animal Care and Use Committee of Xiamen University.

### 2.2. Biochemical Sampling and Analysis

Blood samples were collected from rat hearts under anesthesia after euthanizing. Plasma insulin concentrations were determined by ELISA using commercial kits (Millipore Corporation, USA). Fasting plasma glucose and triglyceride (TG) were measured using commercial assay kits (Nanjing Jiancheng Bioengineering Institute, Jiangsu, China) according to the manufacturer's directions.

### 2.3. Liver Histological Evaluation

The liver tissues in formalin solution were fixed overnight in 10% buffered formalin and embedded in paraffin. The severity of hepatic histologic changes was assessed in hematoxylin-eosin (HE) stained samples and blindly scored by two pathologists who were unaware of the treatments for rats. Steatosis, inflammation, and fibrosis were semiquantitatively evaluated according to Guidelines for Managements of Nonalcoholic Fatty Liver Disease [21]. In NAFLD activity score (NAS), (1) steatosis was scored from 0 to 3 based on a four grades scoring system from S0 to S3, S0: no steatosis or less than 5%, S1: 5–33%, S2: 33–66%, and S3: >66%; (2) lobular inflammation was graded as follows, stage 0: no foci, stage 1: <2 foci per 200x field, stage 2: 2–4 foci per 200x field, and stage 3: >4 foci per 200x field; (3) ballooning degeneration of liver cells was evaluated as follows: grade 0: absent, grade 1: few cells and grade 2: many cells. The histological NAS score was defined as the unweighted sum of the scores for steatosis (0–3), lobular inflammation (0–3), and ballooning degeneration (0–2), thus ranging from 0 to 8. Diagnostic criteria of simple steatosis, borderline NASH, and NASH were based on scores of 0–2, 3-4, and 5 or greater, respectively.

### 2.4. Western Blotting Analysis

Total protein was extracted from liver and skeletal muscle using Protein Extraction Kit (Applygen Technologies Inc., Beijing, China) according to the manufacturer's protocol, respectively. Total protein levels were determined by the bicinchoninic acid (BCA) method (Applygen Technologies Inc., Beijing, China). Equal amounts of protein samples were separated by 10% sodium dodecyl sulphate polyacrylamide gel electrophoresis (SDS-PAGE) and electrotransferred to 0.45 um PVDF membranes. Blotted membranes were blocked with 5% skim milk in TBS with 0.1% Tween 20 and incubated at 4°C overnight, respectively, with one of the following primary antibodies: goat anti-rat adipoR1 polyclonal antibody (diluted to 1 : 1000 with TBS with 0.1% Tween 20; Novus Biologicals, Littleton, CO, USA) or goat anti-rat adipoR2 polyclonal antibody (diluted to 1 : 1000 with TBS with 0.1% Tween 20; Novus Biologicals, Littleton, CO, USA). After three washes in TBS with 0.1% Tween 20, the membranes were incubated with 1 : 5,000 secondary HRP-conjugated anti-goat antibody (MultiSciences Biotech Co., Hangzhou, China) at room temperature for 1 h. Membranes were exposed to the ECL system (Applygen Technologies Inc., Beijing, China) and the bands were quantified with the use of Adobe Photoshop CS5.0 software (Adobe Company, USA).

### 2.5. RT-qPCR Analysis

Total RNA was extracted using Trizol reagent (Invitrogen, SanDiego, CA, USA) from liver and skeletal muscle, respectively, according to manufacturer's instructions. After determination of RNA concentrations by measuring the absorbance at 260 nm and 280 nm, 4 *μ*L RNA as template was reversely transcribed to cDNA by using Revert Aid First Strand cDNA Synthesis Kit (Fermentas, USA). Real-time PCR was performed on 7500 real-time PCR system (ABI Applied Biosystems) using power SYBR Green PCR master Mix. The primer sequences were listed as follows: AdipoR1, forward 5′-GCTGGCCTTTATGCTGCTCG-3′ and reverse 5′-TCTAGGCCGTAACGGAATTC-3′; AdipoR2, forward 5′-CCACAACCTTGCTTCATCTA-3′ and reverse 5′-GATACTGAGGGGTGGCAAAC-3′; *β*-actin, forward 5′-GTAGCCATCCAGGCTGTGTT-3′ and reverse 5′-AACACAGCCTGGATGGCTAC-3′. The cycling conditions were listed as follows: 10 minutes at 95°C as an initial step, 15 seconds at 95°C, and 1 minute at 60°C for 40 cycles for adipoR1/R2. A melting curve analysis was used to confirm specificity of the PCR product, which was demonstrated as a single peak (data not shown). The expression of *β*-actin served as the internal control. Every sample was analyzed in triplicate. A comparative Ct method reported preciously was used in data analysis of real-time PCR.

### 2.6. Statistical Analysis

The variability of results was expressed as mean ± standard deviation. The significance of differences was determined by one-way ANOVA. The difference between two groups was used Student's *t*-test. A two-tailed *P* value of <0.05 was considered statistically significant. SPSS 13.0 for Windows (SPSS Software, Chicago, IL, USA) was used for statistical analysis.

## 3. Results

### 3.1. Liver Pathology

**Figure 1:**
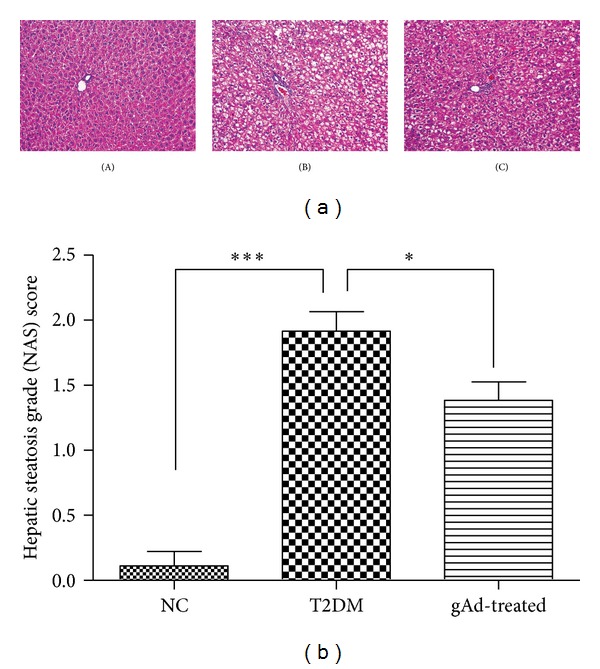
HE staining of liver specimens. (a) HE staining of liver specimens for NC group (A), T2DM group (B), and gAd-treated group (C) (×200). (b) NAFLD activity scores of liver specimens of three groups. *P < 0.05, ***P < 0.001 as compared to T2DM group.

HE staining of liver specimens from T2DM group exhibited steatosis which suggested that the rat model of T2DM was combined with NAFLD. The NAFLD activity score (NAS) of T2DM group was 1.92 ± 0.51, higher than that of NC group (0.11 ± 0.33) (*P* < 0.001). Globular adiponectin treatment made an improvement in the steatosis as compared to T2DM group (1.39 ± 0.51 versus 1.92 ± 0.51, *P* < 0.05) (Figure 1).

### 3.2. Plasma Insulin Level and Glycolipid Metabolism Analysis

**Figure 2:**
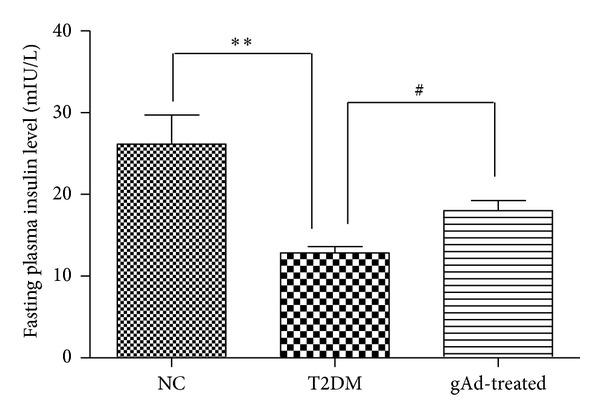
Comparison of plasma insulin levels of all groups.**P < 0.01 compared with NC group, ^#^ *P* < 0.05 compared with T2DM group.

The results of fasting plasma insulin, glucose, and TG were summarized in Table 1. Compared to NC group, fasting plasma insulin level decreased significantly in T2DM group (*P* < 0.01) and it increased in gAd-treated group compared with T2DM group (*P* < 0.05) (Figure 2). The glucose level of T2DM group was higher than NC group (*P* < 0.01), and it was lower in gAd-treated group compared to T2DM group (*P* < 0.01). In addition, the T2DM group had increased level of TG compared with NC group (*P* < 0.01). The concentration of TG in gAd-treated group was lower than T2DM group (*P* < 0.05), which decreased to nearly normal level.

### 3.3. Effects of gAd on AdipoR1/R2 Protein and mRNA Expressions in Liver

**Figure 3:**
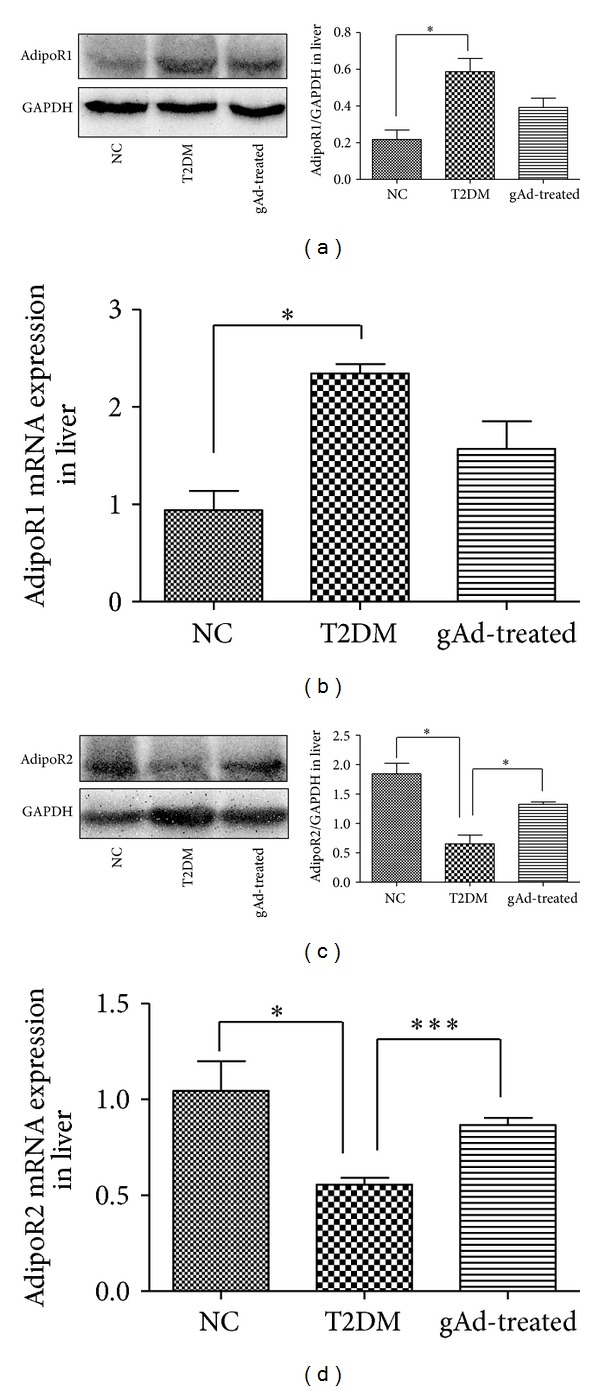
(a) Relative adipoR1 protein expression in liver was determined through western blotting using GAPDH as a reference protein. (b) Relative adipoR1 mRNA expression in rat liver. (c) Relative adipoR2 protein expression in liver was determined through western blotting using GAPDH as a reference protein. (d) Relative adipoR2 mRNA expression in rat liver. *P < 0.05, ***P < 0.001 as compared to T2DM group.

Western blotting analysis in rat liver tissues showed that the expression of adipoR1 increased in T2DM group (*P* < 0.05) and no change was found in gAd-treated group as compared to T2DM group. While the protein expression of adipoR2 in T2DM group decreased significantly in contrast to those of NC group (*P* < 0.05) and increased significantly in gAd-treated group as compared to T2DM group (*P* < 0.05). In addition, the alterations of adipoR1/R2 mRNA expressions were in accord with the alterations of the protein expressions (Figure 3).

### 3.4. Effects of gAd on AdipoR1/R2 Protein and mRNA Expressions in Skeletal Muscle

**Figure 4:**
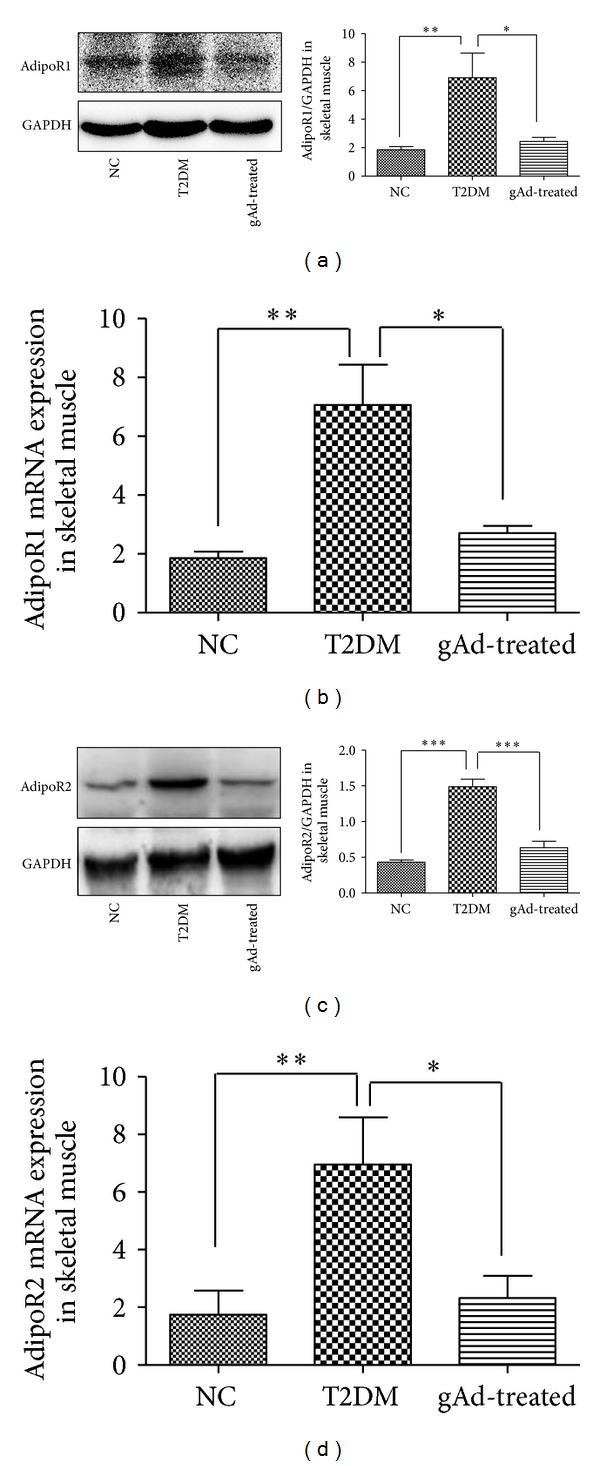
(a) Relative adipoR1 protein expression in skeletal muscle was determined through western blotting using GAPDH as a reference protein. (b) Relative adipoR1 mRNA expression in rat skeletal muscle. (c) Relative adipoR2 protein expression in skeletal muscle was determined through western blotting using GAPDH as a reference protein. (d) Relative adipoR2 mRNA expression in rat skeletal muscle. *P < 0.05, **P < 0.01, ***P < 0.001 as compared to T2DM group.

Western blotting results in skeletal muscle showed that the protein expression of adipoR1 in T2DM group was higher than NC group (*P* < 0.01). Treatment with globular adiponectin significantly downregulated the expressions of adipoR1 protein as compared to the T2DM group rats (*P* < 0.05). The protein expression of adipoR2 also increased in T2DM group compared with NC group (*P* < 0.001) and decreased in gAd-treated group as compared to T2DM group (*P* < 0.001). The alterations of adipoR1/R2 mRNA expressions were in accordance with the alterations of the protein expressions in skeletal muscle (Figure 4).

## 4. Discussion

In our study, high-fat diet and low-dose STZ were used to induce the T2DM rat model. It was close to mimicing the natural history and the metabolic characteristics of type 2 diabetes in humans [22]. The rats exhibited hyperglycemia, together with insulin resistance and deficiency which were in accord with the characteristic of T2DM. In addition, progression of hypoinsulinemia can be detected in severe type 2 diabetic patients clinically at the later stage [23]. The occurrence of NAFLD in T2DM patients is reported to range from 50% to 75% [1, 2]. In our study, we found that the NAS of T2DM group was 1.92 ± 0.51, which was diagnosed as simple steatosis (0–2). Therefore, we induced the T2DM rat model characterized with hypoinsulinemia and hyperglycemia, which mimics T2DM at a later stage closely and is combined with NAFLD characterized with simple steatosis.

Adiponectin is secreted specifically and abundantly in adipose tissue and has anti-inflammatory, antidiabetic, and antiatherogenic properties [4]. It exists as full-length or globular fragment, named full-length adiponectin or globular adiponectin, respectively [9]. The globular adiponectin which had been reported exerted more potent effects [20]. As we know, the specific therapy for T2DM combined with NAFLD was so far lacking. In our study, the recombination globular adiponectin was used for the treatment which had been reported to play an important role in T2DM and NAFLD. It showed that treatment with globular adiponectin reduced hyperglycemia and hypertriglyceridemia of the rats induced by HFD/STZ. Globular adiponectin also stimulated insulin secretion in our experiment. The analysis of liver pathology indicated that globular adiponectin alleviated the steatosis even though it was injected for only one week. The NAS of gAd-treated group decreased significantly as compared to T2DM group (1.39 ± 0.51 versus 1.92 ± 0.51, *P* < 0.05). It indicated that gAd had potential effects on the treatment of T2DM combined with NAFLD.

Liver and skeletal muscles are two vital organs maintaining energy homeostasis with respective mechanism. Skeletal muscle has a fundamentally important role in the maintenance of normal glycolipid homeostasis and in regulating whole-body glycolipid metabolism [24, 25]. Hepatic steatosis may also be associated with the changes of glycolipid metabolism in skeletal muscle.

An evidence supported that globular adiponectin had a higher binding affinity to the membrane fractions of skeletal muscles than full-length adiponectin and had more effects on downstream signaling pathways, whereas only full-length adiponectin did so in the liver [26]. Kadowaki and Yamauchi [12] reported that in skeletal muscle, both globular and full-length adiponectin activated AMPK and then stimulated phosphorylation of ACC, fatty-acid oxidation, and glucose uptake. They also activated PPAR-*α*, thereby stimulating fatty-acid oxidation and decreasing TG content. In the liver, only full-length adiponectin activated AMPK and reduced PEPCK (phosphoenolpyruvate carboxykinase) or G6Pase (glucose-6-phosphatase G6Pase) involved in gluconeogenesis and increased fatty-acid oxidation and decreased tissue TG content just as in skeletal muscle [12]. Our results exhibited that treatment with globular adiponectin reduced hyperglycemia and hypertriglyceridemia induced by HFD/STZ and alleviated the hepatic steatosis. Therefore globular adiponectin may alleviate the hepatic steatosis by improving glycolipid metabolism in skeletal muscle.

The biological functions of adiponectin depend not only on the serum circulating concentration but also on the expression levels and functions of its specific receptors (adipoR1/R2). Some evidences indicated that adipoR1 and adipoR2 were the predominant mediators of the metabolic effects of adiponectin and played an important role in transmitting the signal of adiponectin [26, 27]. AdipoR1 is a high-affinity receptor for globular adiponectin and a low-affinity receptor for full-length adiponectin, while adipoR2 is an intermediate-affinity receptor for full-length and globular adiponectin [18]. But the interaction between adiponectin and adipoR1/R2 still remained controversial. Some evidences supported that adpoR1/R2 were with a similar pattern to transmit the signal of adiponectin [13, 17], while Bjursell et al. [16] observed that adipoR1 and adipoR2 were clearly involved in energy metabolism but had opposing effects. We detected the alterations of adipoR1/R2 in liver and skeletal muscle after globular adiponectin intervention. The results showed that the protein and mRNA expressions of adipoR2 in liver decreased in T2DM group which were in accordance with the report of Beylot et al. [28] and adipoR2 was upregulated after gAd treatment. We also found that the protein and mRNA expressions of adipoR1 were increased in T2DM group and no change was found after gAd treatment as compared to T2DM group. It was postulated that adipoR2 expression decreased in liver and there might be a complementary mechanism which caused the overexpression of adipoR1 in T2DM group. But the fact that the expression of adipoR1 did not change in liver after treatment with gAd might be due to the fact that the dominating tissue which globular adiponectin affected was muscle tissue. Yamauchi et al. [18] found that adipoR1 or adipoR2 enhanced both globular and full-length adiponectin binding, which were associated with increase in PPAR-*α* ligand activity and fatty-acid oxidation. In skeletal muscle, we found that the alterations of adipoR1/R2 differed from the alterations in liver. Both the protein and mRNA expressions of adipoR1 or adipoR2 in T2DM group were higher than NC group and downregulated significantly after globular adiponectin treatment in skeletal muscle. The protein and mRNA expressions of adipoR1/R2 were downregulated after globular adiponectin treatment in skeletal muscle suggesting that it might have a complementary mechanism between liver and skeletal muscle. Some other evidences also supported our results. Inukai et al. [17] reported that adipoR1 mRNA increased in STZ induced diabetic mice, whereas it was reversed by administration of insulin (values for muscle adipoR2 mRNA were not reported). In the vitro experiments of murine C2C12 myotubes cells, Staiger et al. found a trend toward lower adipoR1 mRNA levels with higher insulin concentrations. Staiger et al. [29] also observed that insulin deficiency induced by STZ increased and insulin replenishment reduced the expression of adipoR1/R2 in vivo. Moreover, they found the expressions of adipoR1/R2 in ob/ob mice were significantly decreased in skeletal muscle, which exhibited hyperglycemia and hyperinsulinemia, as compared to control mice [13]. These observations suggested that insulin may negatively regulate the protein and mRNA expressions of adipoR1/R2 in skeletal muscle. In our study, plasma insulin concentration was enhanced after globular adiponectin treatment which was associated with the decreased expressions of adipoR1/R2 in gAd-treated group as compared to T2DM group, suggesting that globular adiponectin may affect the expressions of adipoR1/R2 in skeletal muscle by stimulating insulin secretion.

In conclusion, globular adiponectin varied the expressions of adiponectin receptors in liver and skeletal muscle. the fact that it decreased the expressions of adipoR1/R2 in skeletal muscle might be due to the stimulation of insulin secretion. Globular adiponectin may ameliorate the hepatic steatosis by stimulating insulin secretion and improving glycolipid metabolism in skeletal muscle. Globular adiponectin might be considered as a potential therapy for T2DM combined with NAFLD.

## Figures and Tables

**Table 1:** The fasting plasma insulin, glucose, and TG levels of rats ($\overset{-}{x}\pm s$).

	NC group (*n* = 7)	T2DM group (*n* = 7)	gAd-treated group (*n* = 7)
Insulin (mIU/L)	26.2 ± 9.5	12.8 ± 1.8**	18.0 ± 3.2^#^
Glucose (mmol/L)	7.1 ± 0.8	24.8 ± 1.3**	20.9 ± 1.9^##^
TG (mmol/L)	1.3 ± 0.1	1.8 ± 0.2**	1.4 ± 0.2^#^

**P < 0.01 compared with NC group, ^#^ *P* < 0.05, ^##^ *P* < 0.01 compared with T2DM group.

## References

[1] Targher G, Bertolini L, Padovani R (2007). Prevalence of nonalcoholic fatty liver disease and its association with cardiovascular disease among type 2 diabetic patients. *Diabetes Care*.

[2] Williamson RM, Price JF, Glancy S (2011). Prevalence of and risk factors for hepatic steatosis and nonalcoholic fatty liver disease in people with type 2 diabetes: the Edinburgh type 2 diabetes study. *Diabetes Care*.

[3] Targher G, Byrne CD (2013). Clinical Review: nonalcoholic fatty liver disease: a novel cardiometabolic risk factor for type 2 diabetes and its complications. *Journal of Clinical Endocrinology and Metabolism*.

[4] Kadowaki T, Yamauchi T, Kubota N, Hara K, Ueki K, Tobe K (2006). Adiponectin and adiponectin receptors in insulin resistance, diabetes, and the metabolic syndrome. *Journal of Clinical Investigation*.

[5] Hivert MF, Sullivan LM, Fox CS (2008). Associations of adiponectin, resistin, and tumor necrosis factor-*α* with insulin resistance. *Journal of Clinical Endocrinology and Metabolism*.

[6] Arita Y, Kihara S, Ouchi N (1999). Paradoxical decrease of an adipose-specific protein, adiponectin, in obesity. *Biochemical and Biophysical Research Communications*.

[7] Li S, Shin HJ, Ding EL, Van Dam RM (2009). Adiponectin levels and risk of type 2 diabetes: a systematic review and meta-analysis. *Journal of the American Medical Association*.

[8] Kubota N, Terauchi Y, Yamauchi T (2002). Disruption of adiponectin causes insulin resistance and neointimal formation. *Journal of Biological Chemistry*.

[9] Fruebis J, Tsao T, Javorschi S (2001). Proteolytic cleavage product of 30-kDa adipocyte complement-related protein increases fatty acid oxidation in muscle and causes weight loss in mice. *Proceedings of the National Academy of Sciences of the United States of America*.

[10] Kim JY, van de Wall E, Laplante M (2007). Obesity-associated improvements in metabolic profile through expansion of adipose tissue. *Journal of Clinical Investigation*.

[11] Berg AH, Combs TP, Du X, Brownlee M, Scherer PE (2001). The adipocyte-secreted protein Acrp30 enhances hepatic insulin action. *Nature Medicine*.

[12] Kadowaki T, Yamauchi T (2005). Adiponectin and adiponectin receptors. *Endocrine Reviews*.

[13] Tsuchida A, Yamauchi T, Ito Y (2004). Insulin/Foxo1 pathway regulates expression levels of adiponectin receptors and adiponectin sensitivity. *Journal of Biological Chemistry*.

[14] Debard C, Laville M, Berbe V (2004). Expression of key genes of fatty acid oxidation, including adiponectin receptors, in skeletal muscle of type 2 diabetic patients. *Diabetologia*.

[15] Yamauchi T, Nio Y, Maki T (2007). Targeted disruption of AdipoR1 and AdipoR2 causes abrogation of adiponectin binding and metabolic actions. *Nature Medicine*.

[16] Bjursell M, Ahnmark A, Bohlooly-Y M (2007). Opposing effects of adiponectin receptors 1 and 2 on energy metabolism. *Diabetes*.

[17] Inukai K, Nakashima Y, Watanabe M (2005). Regulation of adiponectin receptor gene expression in diabetic mice. *American Journal of Physiology*.

[18] Yamauchi T, Kamon J, Ito Y (2003). Cloning of adiponectin receptors that mediate antidiabetic metabolic effects. *Nature*.

[19] Ceddia RB, Somwar R, Maida A, Fang X, Bikopoulos G, Sweeney G (2005). Globular adiponectin increases GLUT4 translocation and glucose uptake but reduces glycogen synthesis in rat skeletal muscle cells. *Diabetologia*.

[20] Yamauchi T, Kamon J, Waki H (2001). The fat-derived hormone adiponectin reverses insulin resistance associated with both lipoatrophy and obesity. *Nature Medicine*.

[21] Fan JG, Jia JD, Li YM (2011). Guidelines for the diagnosis and management of nonalcoholic fatty liver disease: Update 2010: (Published in Chinese on Chinese Journal of Hepatology 2010; 18:163–166) JG Fan etal. Diagnosis and management of NAFLD. *Journal of Digestive Diseases*.

[22] Zheng XK, Zhang L, Wang WW, Wu YY, Zhang QB, Feng WS (2011). Anti-diabetic activity and potential mechanism of total flavonoids of Selaginella tamariscina (Beauv.) Spring in rats induced by high fat diet and low dose STZ. *Journal of Ethnopharmacology*.

[23] Mathis D, Vence L, Benoist C (2001). *β*-cell death during progression to diabetes. *Nature*.

[24] Sinacore DR, Gulve EA (1993). The role of skeletal muscle in glucose transport, glucose homeostasis, and insulin resistance: implications for physical therapy. *Physical Therapy*.

[25] Hulver MW, Berggren JR, Cortright RN (2003). Skeletal muscle lipid metabolism with obesity. *American Journal of Physiology*.

[26] Yamauchi T, Kamon J, Minokoshi Y (2002). Adiponectin stimulates glucose utilization and fatty-acid oxidation by activating AMP-activated protein kinase. *Nature Medicine*.

[27] Myeong JY, Gha YL, Chung J, Young HA, Seung HH, Jae BK (2006). Adiponectin increases fatty acid oxidation in skeletal muscle cells by sequential activation of AMP-activated protein kinase, p38 mitogen-activated protein kinase, and peroxisome proliferator-activated receptor{alpha}. *Diabetes*.

[28] Beylot M, Pinteur C, Peroni O (2006). Expression of the adiponectin receptors AdipoR1 and AdipoR2 in lean rats and in obese Zucker rats. *Metabolism*.

[29] Staiger H, Kaltenbach S, Staiger K (2004). Expression of adiponectin receptor mRNA in human skeletal muscle cells is related to in vivo parameters of glucose and lipid metabolism. *Diabetes*.

